# Sulfidogenic fluidized-bed bioreactor kinetics for co-treatment of hospital wastewater and acid mine drainage

**DOI:** 10.1016/j.btre.2021.e00683

**Published:** 2021-10-14

**Authors:** Thobeka Pearl Makhathini, Jean Mulopo, Babatunde Femi Bakare

**Affiliations:** a School of Chemical and Metallurgical Engineering, University of the Witwatersrand, P/Bag 3, Wits 2050, Johannesburg, South Africa; b Department of Chemical Engineering, Mangosuthu University of Technology, 511 Mangosuthu Highway, Umlazi, Durban 4031, South Africa

**Keywords:** Cod oxidation, Kinetics, Sulfate-reducing bacteria, Hospital wastewater

## Abstract

A passive co-treatment of acid mine drainage and hospital wastewater previously demonstrated a promising bioremediation viable approach for both toxic streams. The study of inhibition kinetics and microbial communities is essential to understand better the diverse species and the reaction mechanisms within the system. The kinetics and microbiology diversity in the sulfidogenic fluidized-bed reactor (at 30 °C) for co-treatment of hospital wastewater and metal-containing acidic water were examined. The alkalinity from organic oxidation raised the pH of the effluent from 2.3 to 6.1–8.2. Michaelis-Menten modeling yielded (*K*_m_ =7.3 mg/l, *V*_max_ = 0.12 mg/l min^−1^) in the batch bioreactor treatment using sulfate-reducing bacteria. For COD oxidation, the dissolved sulfide inhibition constant (K_i_) was 3.6 mg/l, and the K_i_ value for H_2_S was 9 mg/l. The dominant species in the treatment process belong to the *Proteobacteria* group (especially *Deltaproteobacteria*). The ibuprofen and diclofenac compounds achieved the highest removal rates in the bioreactor of 58.6% and 52.3%, respectively; while, ketoprofen and naproxen of 41.9% and 46.6%, respectively. The findings in COD kinetics, sulfate-reducing bacteria abundance, and selected pharmaceutical concentration reduction provide insight into this co-treatment process's capability.

## Highlights

• Bioremediation process for acidic mine water co-treatment with hospital wastewater.
• Metal precipitation reached 98% and soluble concentrations of Fe and Zn were less than 0.1 mg/l.
• SO_4_^2−^ removal was above 90% in the sulfidogenic bioreactor.
• Naproxen, ibuprofen, ketoprofen, and diclofenac partially removed during the co-treatment process.

## Graphical abstract

**Figure:**
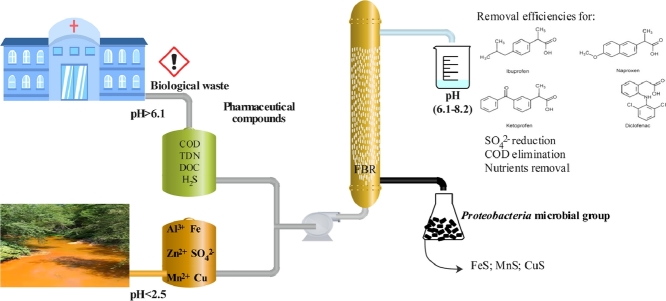

## 1 Introduction

Treatment of wastewater from mineral processing and mining still demands an alternative to predictable chemical treatments, which is usually expensive. Amongst, alternative treatment options are the sulfate-reducing bioreactors [1] and co-treatment processes. The process of bioremediation is reliant on hydrogen sulfide (Eq. (1)) using sulfate-reducing bacteria (SRB) in an anaerobic environment.

(1) $$S{O_{4}}^{2-}+2CH_{2}O\left(\text{electron}\text{donor}\right)\;\to H_{2}S+2\text{HC}{O_{3}}^{-}$$

Then, alkalinity production (Eq. (2)) through the electron donor's oxidation while precipitating metal sulfide.

(2) $$\text{HC}{O_{3}}^{-}+H^{+}\to \;CO_{2}+H_{2}O$$

Usually, the bioremediation process option requires an electron donor for sulfate-reducing bacteria (SRB), which becomes a drawback for the process as it involves high operational costs. Hence, it is crucial for researchers to continuously seek ways of replacing commercial electron donors with low-cost electron donor options. The treatment option may include mixing acid mine water with another stream like fermentation industry wastewater or landfill leachate to benefit the mixed streams regarding pollution reduction. For example, Roetman [2] was the first to propose to mix acid mine waster and municipal wastewater to reduce pathogens in sewage. After that, more studies were developed to assess the practicability of the co-treatment approaches in acid mine drainage (AMD) remediation and reduction of organic matter from wastewater [3], [4], [5]. These studies demonstrated improvements in water quality for metal, organics, and nutrients removal with elevated pH and alkalinity. As such, this study hypothesized that any other waste stream that has elevated concentration of COD and nutrients is likely to produce higher sulfate reduction in a passive co-treatment with AMD.

On the other hand, pharmaceuticals are increasingly detected in surface waters, and drinking water as not all are removed in traditional wastewater treatment plants [6]. Hospital wastewater can be considered one of the point sources of pharmaceuticals, and separate treatment of this waste stream is of interest. One of the treatment options of pharmaceuticals in wastewater is degradation through biological remediation, including bioreactors. To date, only one bacterial strain, which degrades ibuprofen and uses ibuprofen as a carbon and energy source, has been described, although less is confirmed by the bacteria that degrade these compounds and their underlying biodegradation mechanisms [7]. Diclofenac is biologically degradable, yet the bacteria responsible are unidentified.

The ability to directly utilize hospital wastewater is not a common feature of the SRB. The combination of acid mine water in hospital wastewater treatment promises great environmental merits compared to traditional activated sludge processes achieved by combining the two stream's water chemistry. For example, a high sulfate concentration in AMD can function as an SRB electron-acceptor to oxidize organic compounds Deng et al. [8] in hospital wastewater (HWW) under anaerobic conditions. In this case, the active treatment method, which requires energy consumption in wastewater treatment plants, is eliminated [9]. Also, biological sludge production is reduced significantly under anaerobic conditions [10].

The biological approach of treating AMD is derived from various microorganisms' potential to generate alkalinity, eliminate the metals, and then reverse the reactions responsible for AMD formation [11]. The available carbon and electron source for SRB can support the biological approach utilizing sulfate reduction. Wastewater treatment processes that utilize sulfate-reducing bacteria include reactive barriers, wetlands, and bioreactors [10]. Unlike other bioreactors, fluidized bed reactors (FBRs) are superior in terms of less clogging or channeling, excellent treatment efficiencies, and a low probability of shock loads [12].

A fluidized bed reactor achieves better sulfate reduction rates and greater carrier surface area than anaerobic filter reactor [13]. In an FBR, carrier material assists with settable biomass through biofilm development in comparison to granulation in an up-flow anaerobic granular sludge bed (UASB) [14]. Moreover, effluent recycling, like the extended granular sludge bed (EGSB) reactor, results in the carrier material's fluidization. The sulfate-reducing FBRs for rehabilitating metal-containing acidic wastewater using sand as a carrying medium has been used [15].The reduction of biological sulfate is performed under mesophilic conditions (25–35 °C), even under thermophilic conditions (35–70 °C) [16]. The thermophilic process for reducing sulfates is suitable for treating reasonably warm metal-containing water, such as the wastewater from the pulp and paper industry. As such, this study uses hospital wastewater, so it is fitting to assess the biotreatment at mesophilic conditions.

The co-treatment system has important aspects to be considered such as mixed water chemistry, reactor configuration, COD/SO_4_^2−^ ratios and microbiological diversity [10]. Despite being thought to play a significant impact in treatment success, little is known about microbial ecology and its interactions with co-treatment kinetics. An earlier study reported AMD co-treatment process from an abandoned mine and an isolated stream of hospital wastewater, mixing the streams then pumped to a sulfidogenic bioreactor, showed effective reduction rates of COD, sulfate, metals, and selected pharmaceutical compounds [17]. This research study is focused on sulfide kinetics, iron inhibitory effects, and the fluidized-bed reactor's microbial ecology. Largely, the iron inhibitive effects are likely to differ depending on reactor configuration, SRB species, metal concentration, pH, and E_h_ conditions. The study presents the first attempt to assess the degradability of the selected anti-inflammatory pharmaceutical compounds in the sulfidogenic FBR co-treatment system, that is, ibuprofen, diclofenac, ketoprofen, and naproxen.

## 2 Materials and methods

### 2.1 Sampling

Acid mine water was collected at the abandoned mining site in Mpumalanga Province, South Africa. Hospital wastewater (HWW) was collected at the effluent point from a public hospital in Kwa Zulu Natal, South Africa. A sampling of HWW was carried out at regular intervals from August 2019 to January 2020, collecting 500 ml per time and contained in 4 °C cooled acid-washed bottles to check the variability of constituents in HWW samples in this site. During the sample collection trips, on-site measurements of pH, temperature, and electrical conductivity were tested. Municipal wastewater samples are collected from a southern Durban treatment plant, in South Africa, for the same duration as the HWW. Key constituents in HWW and AMD samples are shown in Table 1.

**Table 1:** Key constituents in acid mine drainage, hospital wastewater and municipal wastewater.

Characteristics	HWW Range	AMD
pH	6.1–8.3 ± 0.4	2.34
Alkalinity	412 ± 45	0
COD (mg l^−1^)	136–15,788 ± 116	22
DOC (mg l^−1^)	45–235 ± 102	< 1
TDN (mg l^−1^)	212–1445 ± 12	< 1
H_2_S (mg l^−1^)	0.092 ± 1.1	< 0.001
SO_4_^2−^ (mg l^−1^) PO_4_^3−^ (mg l^−1^)	34–49 ± 1.8 11.6–37 ± 0.4	3212 < 0.2
Cl^−^ (mg l^−1^)	145–22 ± 0.5	< 3
Fe (mg l^−1^)	< 0.03	1305
Mn (mg l^−1^)	< 0.07	102
Al (mg l^−1^)	0.11	218
Cu (mg l^−1^)	< 0.01	25
Zn (mg l^−1^)	< 0.01	96

### 2.2 The biotreatment protocol

**Fig. 1:**
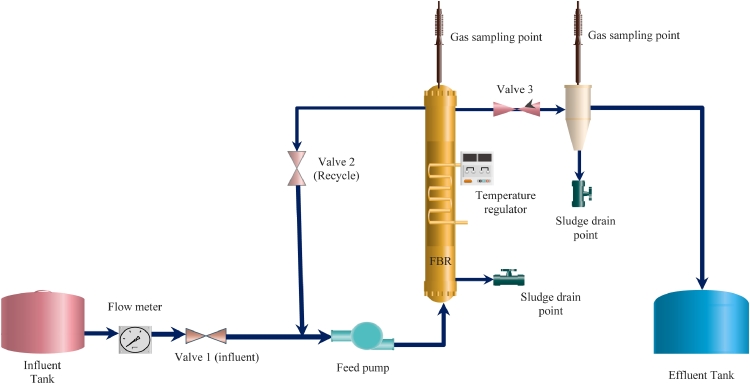
Configuration of the fluidized-bed reactor.

A fluidized-bed reactor was used for this treatment system with 2000 ml (Fig. 1). Each FBR was inoculated with 200 ml of anaerobic sludge harvested from the Durban South Basin wastewater treatment plant, after packing with silica sand (Spec Silica Sand, SA: medium loading) 20% fluidization rate for biofilm development. Also, the study observed one reactor with HWW only and another with AMD only for control experiments. The control bioreactors were only sampled on the last day of the experiment. The biological treatment experiments were run for six months at approximately 30 °C using a heating element to control the temperature, where physiochemical, nutrient, organic carbon parameters, and metals were continuously monitored. During the initial phase of the process and the loading, experiments took 60 days to enrich the SRB. On day 180, sludge was sampled for chemical element analysis. The control experiments were run in parallel reactors to assess the abiotic process contribution to sulfate and COD removal.

**Fig. 2:**
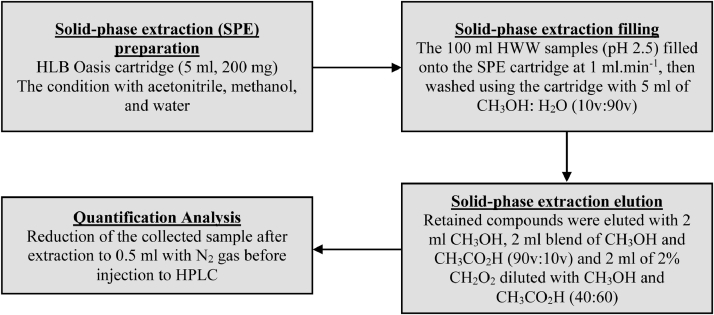
Changes in the hydraulic retention time (HRT) in the FBR operation.

Since the previous study had provided some insight into the co-treatment influence; the hydraulic retention time (HRT) was varied as per previously investigated values [17]. The HRT in this experiment was gradually dropped from 24 to 16, 12, and finally to 4 h (Fig. 2), though the feed rate remained relatively constant. Whilst real AMD and HWW effluent samples were used without any pre-treatment. However, a stage where the COD of the HWW was low, and meat extract was added to increase the concentration to maintain the COD/SO_4_^2−^ ratio as such influent flow rate was varied depending on the targeted HRT value.

Meanwhile, batch kinetics experiments were carried out in intermittent stages during the biotreatment processing to find the kinetic factors (*V*_max_ and *K*_m_) for the oxidation of COD in HWW. When the reactor was running on continuous mode, it was continually fed at HRT of 24 h, (as the peaks are seen in Fig. 2) with HWW only, until the next batch experiment was carried out. The reactor was taken back to run at 24 h HRT to stabilize the system after a process disturbance occurred. This procedure ensures the cleanout of excess H_2_S accumulated through the batch experiments and retains constant biomass in the FBR [15]. During each batch experimental run, the influent flow was stopped, and the FBR was controlled in a recycling operation. In each batch experiment's initial and final period, sulfide and sulfate samples were collected for analysis.

### 2.3 Chemical analysis

All samples obtained in the field were filtered using 0.4 microfilters before being analyzed. Temperature, conductivity, and pH were measured using a calibrated PHS-3BW pH meter maintained according to the supplier's recommendations. The auto-titrator Thermo Science Orion Star T900 was used to determine the alkalinity and acidity of the samples. The unfiltered samples were tested for COD using the Hach DR3900 spectrophotometer and the APHA [18] standard techniques. Filtered samples were tested for NH_4—_N, sulfide, sulfate, and metals using APHA [18] standard techniques.

COD, sulfate, and sulfide were measured using a Hach DR3900 spectrophotometer. The sample cell was filled with 10 mL deionized water for the sulfide measurement. Then, in a second sample cell, 10 mL of sample is added and properly stirred to prevent sulfide loss. Each sample cell received a pipette of 0.5 ml sulfide reagent, which was thoroughly stirred. The sample cells are then inverted to mix and left to react for five minutes with 0.5 ml of sulfide reagent two added to each cell. The blank cell is used to reset the instrument (DR3900) after the time-lapse, and then the sample cell is read.

The DRB200 was prepared for COD concentration measurement by preheating the temperature to 150 °C. While waiting for the instrument to warm up, a 100 ml sample is homogeneously blended for 30 to 60 s in a blender. 0.3 ml of sample was pipetted into the TNT plus vials, then the vial was inverted several times for optimum mixing. After that, the vials were placed in a sealed, preheated DR3900 reactor for two hours. Following the time lapse, the vials were removed from the reactor, cooled to ambient temperature, and then put into the cell holder to obtain a reading. Hach standards procedures were used to determine the amount of dissolved organic carbon (DOC).

For sulfate measurements, the sample cell was filled with 10 ml of sample, then a SulfaVer 4 powder pillow was added to completely dissolve the powder. After that, the cell was left for five minutes to finish the reaction. After the time-lapse, the DR3900 was reset by inserting a blank sample cell into the cell holder, and then the prepared sample was introduced into the cell holder to read the results [19].

300 ml incubation bottles and a nitrifying inhibitor were required for the BOD measurement apparatus (N-Allylthiourea). A 50–100 ml volume of sample was poured to the incubation bottles, followed by a few drops of ATH, and then the BOD system was placed in an incubator at 200 C for five days. To determine the BOD, the dissolved residual oxygen for all investigated samples was measured after five days.

The total dissolved nitrogen (TDN) measurements were initiated by preheating the DRB200 reactor to 103 °C. A total nitrogen persulfate reagent powder pillow was added to two high range total nitrogen hydroxide digestion reagent vials. After that, 0.5 ml of sample was added to one of the vials, and the other vial, which was 0.5 ml of deionized water, was added. The prepared vials were put in the reactor for 30 min; after time elapsed, vials were cooled down to room temperature before second total nitrogen (TN) reagent A was added. A 3 min reaction took place, then TN reagent B was added, allowing a further 2 min for the reaction. On both the prepared sample and on the blank, a 2 ml TN reagent C was added. A 5 min reaction time was allowed then samples were read on the spectrophotometer.

Volatile suspended solids (VSS) analysis was done is influent and effluent samples through filtering 50 ml using a Whatman fiber filter, thereafter, drying the filter for 60 min in a preheated drying oven at 103 to 105 °C, lastly igniting for further 15 min in a preheated furnace at 550 °C. Finally cool off the sample and weight the sample. The residue (VS) was determined by comparing the mass of the sample before and after each drying step.

For all dissolved samples, the pH was kept at < 2 using concentrated HNO_3_, then stored at 4 °C till used. Syringe filters with a 0.45 nylon membrane were used to prepare the samples for metal concentration analysis of Al, Fe, Cu, Mg, Pb, Mn, and Zn before injecting into ICP-OES (Varian 720-ES). The analysis was performed in duplicates, as recommended by the USEPA protocol.

### 2.4 Kinetics modeling

The COD oxidation rates were conducted during batch kinetic experiments and standardized to the total amount of biomass in the bioreactor. The accumulation of biomass inside the fluidized-bed reactor was estimated using Eq. (3) where B_FBR_ is the amount of biomass (mg VS) accumulated inside the reactor; VS_y_ and VS_x_ is the volatile solids at the end and at the beginning of the experiment, respectively; V_c_ volume of the carrier (l);$\rho_{c}$ is the density of the carrier material (g/l). The biomass yield was estimated using Eq. (4), where B_yield_ is the biomass yield (mg) in the reactor, and Bout is the biomass washout (mg) as volatile suspended solids (VSS) [15].

(3) $$B_{FBR}=VS_{y}V_{c}\rho_{c}-VS_{x}$$

(4) $$B_{yield}=B_{FBR}+B_{out}$$

Estimation of maximum reaction rate (*V*_max_, mg/l) and constant of Michaelis-Menten (*K*_m_, mg/l) (Eq. (5)) using the Lineweaver-Burk transformation equation [15,20]:

(5) $$\frac{1}{V}=\frac{1}{V_{max}}+\frac{K_{m}}{V_{max}}\times \frac{1}{S}$$

where S is the substrate concentration, COD (mg/l), and *V* is the rate of reaction (mg/l min). The inhibition constants (K_i_) were estimated through Fe concentrations using the noncompetitive (Eq. (6)) inhibition model [20] for H_2_S and DS.

(6) $$V=\frac{V_{max}\times S}{\left(K_{m}+S\right)\times \left(1+\frac{1}{K_{i}}\right)}$$

Where *I*= concentration of inhibitor (mg/l), and constant inhibition of K_i_ (mg/l). The model was fitted to the data to estimate *K*_i_, *V*_max,_ and *K*_m_ using a non-linear least-squares optimization subroutine, MATLAB_R2020a.

The dissolved (total) sulfide concentration was used to calculate the undissociated hydrogen sulfide concentration from the dissociation constant (*K*_a1_) of H_2_S using (Eq. (7)).

(7) $$H_{2}S=\frac{DS}{1+10^{pH-pK_{a1}}}$$

where p*K*_a1_ is -log *K*_a1_.

### 2.5 Microbiological analyze

The RNA (total) was extracted from the sludge with modifications by RNA isolation kit (Merck, SA). The samples were diluted with 10 mL of the reagent from the RNA isolation kit. The samples were suspended and subjected to ultrasonic vibration to fragment the cells and macromolecules on ice using ultrasonic generator (Hielscher, Germany) for three minutes. Samples were mixed with 2.4 ml of chloroform. For five minutes, samples were placed on ice, then centrifuged for fifteen minutes at 4 °C. The method by Lin and Stahl [21] was used to purify the rRNA extracts. The rRNA extracts were suspended again in a lysis buffer.

The microbial FBR communities were evaluated using clone libraries and denaturing gradient gel electrophoresis (DGGE) of the polymerase chain reaction of 16S rRNA genes [22]. The dsrA gene associated with sulfate-reduction was quantified by polymerase chain reaction (qPCR) analyzes [23]. The oligonucleotides probes targeting the 16S rRNA-gene of anaerobic bacteria were used [22]. Amplified 16S rRNA-gene sequences were cloned and analyzed phylogenetically as prescribed by Jeon et al. [24].

### 2.6 Ecotoxicological analysis

Toxicity was determined by standard toxicity tests, *Vibrio fischeri* [25], and *Daphnia magna* [26]. The first test (*D. magna*) was conducted using neonates hatched at 20–22 °C for 72 h, under illumination conditions. Each sample was analyses using replicates, ten neonates were used in each dilution with *D. magna*, and dilutions were incubated at approximately 20 °C. Then intermittently between 24 and 48 h in the incubator, the organisms were measured. Instead, with V. *fischeri*, each sample dilution's osmolality was corrected to achieve a 2% saline. The units of toxicity were determined according to the Sprague and Ramsay [27] equation, as shown in Eq. (8).

(8) $$\text{TU}\;=\;{\left(EC_{50}\right)}^{-1}\times 100$$

Each sample dilution was tested in replicate, and the sample concentration assigned as EC_50_ at 15 °C exposure after 30 min, resulting in a 50 percent bioluminescence inhibition.

### 2.7 Pharmaceutical analysis

**Fig. 3:**
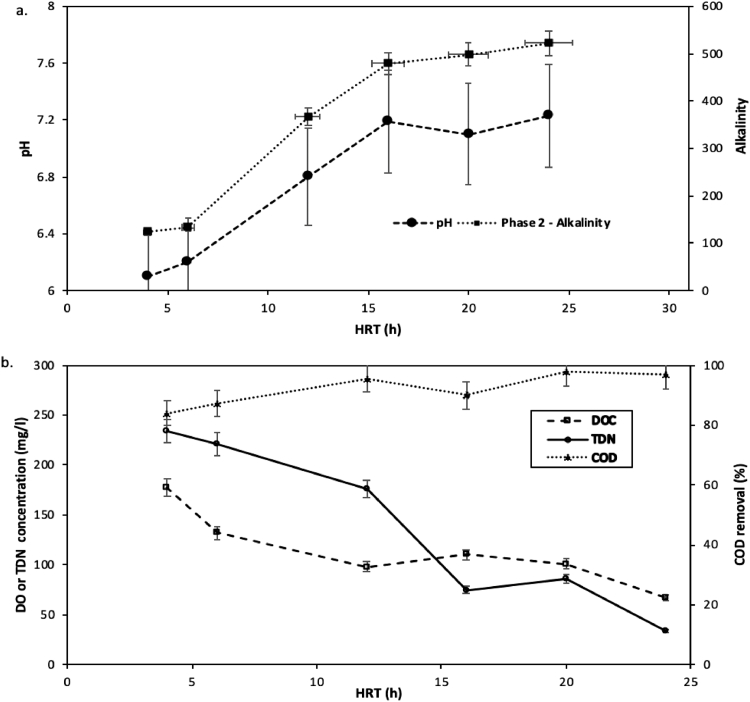
The protocol flow diagram which summarizes the steps involved in pharmaceuticals analysis.

Sigma-Aldrich (Germany) supplied ibuprofen (> 98%), naproxen (98%), ketoprofen and diclofenac sodium salt used for quantification analysis. Ethyl acetate (> 99.9%), methyl alcohol (99.5%), acetonitrile (> 99.9%), and acetone (99.5%) were solvents of high-pressure liquid chromatography grade from Macron Fine Chemicals, SA. Analytical grade was also used for specific reagents. The solid-phase extraction (SPE) cartridges used were 5 ml (200 mg mass) Oasis HLB. The protocol adopted in this study to measure ibuprofen, diclofenac, ketoprofen, and naproxen was adapted following the modification of the methods proposed by Agunbiade and Moodley [6]. Fig. 3 provides an overview of the technique.

The process has been validated by evaluating the efficiency of solid-phase extraction (1 μg. l^−1^), the detection limits, and linearity. Detection limits found three times the average four-sample deviation (blank), and linearity was based on the coefficient (r^2^). Finally, effluent tests in the bioreactor system were used instead of deionized water [28]. Considering the initial concentration of the hospital wastewater sample (C_i_), the influential concentration at day 0 (C_0_), and the final effluent at day 30 (C_f_), disposal efficiencies from the treatment method were determined. The percentage of phase 2 reactor elimination was obtained by [C_f_-C_0_]/C_i_ and the overall output using [C_i_ – C_f_]/C_i_. For all cases, C_i_ is used as a guide for comparison and the average amount for receiving.

## 3 Results and discussion

### 3.1 Co-treatment evaluation

**Fig. 4:**
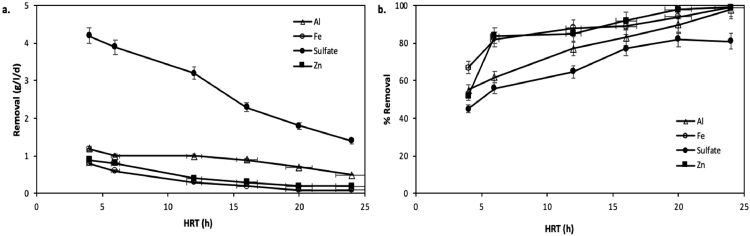
(a) pH and alkalinity dynamic response over a 180-day co-treatment period, at different HRT (b) Total dissolved nitrogen (TDN), DOC and COD concentrations at the end of day 180-day treatment period, at different HRT.

After aerobic mixing of the samples, the remediation HWW and AMD started with the chemical phase, leading to an increase of 2.5 AMD pH to 6.1–8.2 (Fig. 4a). After a few hours of mixing, precipitates of metal hydroxides emerged in all reactors due to the coagulant properties of metal cations [29]. The dissolved oxygen and alkalinity only experienced a change as time passed, which is associated to the chemical reactions related to aerobic mixing preceded by sulfate reduction [30]. The initial phase of aerobic mixing resulted in net alkaline conditions after mixing, and even in the second phase of biological treatment, further alkalinity was produced. A slight pH decreased from 7.19 ± 0.10 to 7.10 ± 0.02 between 4 and 8 h HRT in the treatment system, then recovered to 7.23 ± 0.14 by 20 h HRT (checked by one-way ANOVA, where *F* = 6.11 *P* < 0.001). This trend was expected to show in earlier days, which could have been attributed to metal hydroxides.

Nonetheless, the pH recovery increase is attributed to high bicarbonate production by reducing sulfate. The pH trend followed the alkalinity production from 479 ± 11 to 498 ± 8 mg/l at 16 h and 20 h HRT, respectively, thereafter remained almost the same for 24 h HRT. The production of alkalinity may be attributed to the biotic reduction of sulfate to hydrogen sulfide, thus generating bicarbonate ions [11].

Nitrogen and organic carbon concentrations were almost certainly influenced by precipitation in the initial phase of the experiment. An overall observation of BOD, DOC, and TDN proved a decrease over the 180 days duration. It is most likely that the bacterial sulfate reduction facilitated the efficiency of processing the BOD and DOC [9]. The HWW control sample demonstrated about 28% and 37% decrease in DOC and COD, in that order. The inorganic nitrogen in the reactors was introduced from the HWW sample in ammonia form and measured a 9–28% NH_4_^+^ decline at the end of the 180-day treatment period. The DOC concentrations followed the Fe concentration decline trend, apart from 16 h HRT, where there was a slight increase to 110 ± 11 mg/l (Fig. 4b). Nitrate and nitrite concentrations were recorded to be reasonably low at ≤0.1 mg/l and ≤ 5μg/l, in that order.

**Fig. 5:**
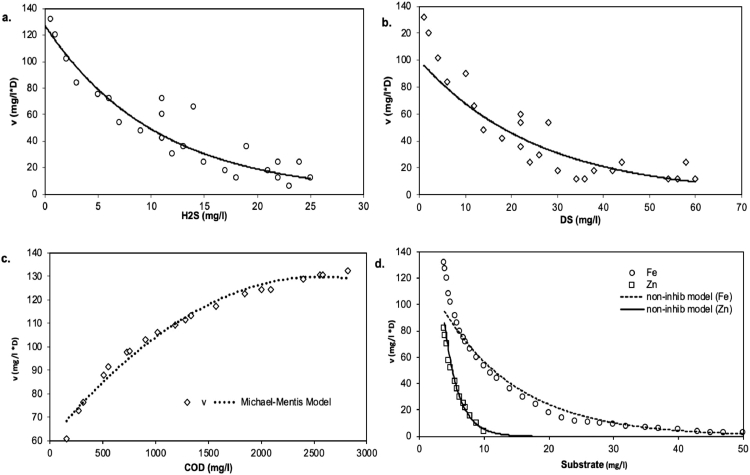
The effects of hydraulic retention time on (a) amount of sulfate removed and (b) removal rate of sulfate, Al, Fe and Zn in the FBR.

The initial phase of the experiment, where HWW and AMD were aerobically mixed, Al and Fe recorded an efficient removal from the rest of the metals. In distinctive reactors, as per AMD/HWW mixing ratio, the concentrations of metals differed as such. Compared to control samples, the combined treatment of HWW and AMD demonstrated outstanding reduction rates, (Al: > 99%, Fe: > 98%, Mg: 44–65%, Mn> 87%, and Zn: 52–79%) which is the highest removal recorder rate of manganese thus far from co-treatment studies. Further reduction of Al and Fe in biological treatment is most likely facilitated by AMD's combination with organic binding molecules [31]. On 12 h HRT (Fig. 5), the treatment measured an increase in Fe concentration, which may well be due to microbial Fe decrease of FePO_4_ and Fe(OH)_3_ [9]. Iron sulfide precipitated due to the sulfate-reducing environment dominant at this experiment [32]. Lastly, metal biosorption to the organic molecules related to the metabolism mechanism may cause metal removal [8,9,[33], [34], [35]]. The high COD removal rate and decrease in TDN and BOD confirm the removal of dissolved nitrogen and organic carbon during the 180-day treatment period, only a small amount of concentration remained at the end of the treatment.

### 3.2 Modeling and COD oxidation kinetics

The COD, BOD, and TDN concentration decrease were accompanied by biomass accumulation in the reactors during the 180-day treatment period. The total biomass quantity in the rector was approximately 3.8 ± 0.81 g during the oxidation period. On average, 87–90% of the biomass was attached to the silica sand material, 7–8% on metal precipitates not attached to the carrier material, and 4–5% may be found in the effluent stream [36].

**Fig. 6:**
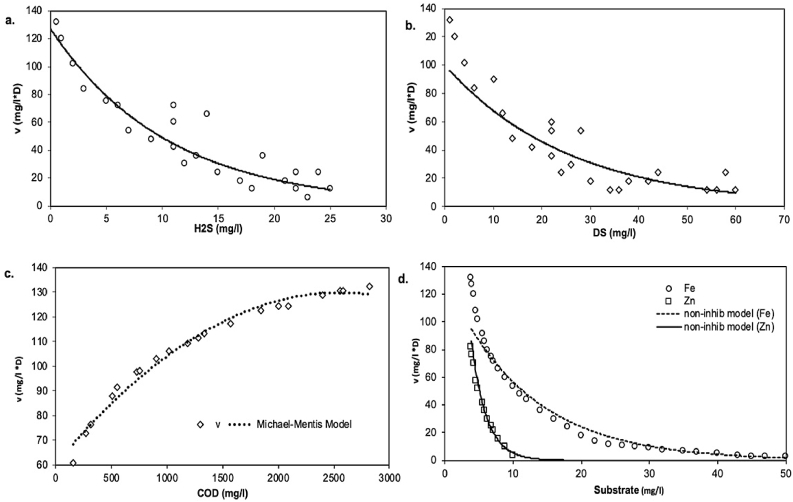
The effect of (a) hydrogen sulfide (H_2_S) and (b) dissolved sulfide (DS) in the batch reactor experiments. The noncompetitive inhibition model fit is represented by the curves. Michaelis-Menten and inhibition model for sulfidogenic COD oxidation rate (*V*), and (b) Fe and Al non-competitive inhibition model.

Fig. 6a and b demonstrate the impact of concentrations of dissolved sulfide and hydrogen sulfide on COD oxidation levels. The Michaelis-Menten (*K*_m_) constant and maximum oxidation of COD (*V*_max_) were assessed as 42 mg/l and 0.12 mg/l min, respectively (Fig. 6a and b). The *K*_m_ was valued was less compared to the study that used MWW as a source of SRB [8], where an addendum to Deng et al. [8] study was published in 2018, with *K*_m_ changed to 6220 mg/l but still higher than the other studies that used pure SRB cultures [37]. This current study achieved a *K*_m_ value comparable to *Desulforhabdus amnigenus* and *Desulfobacca acetoxidans* culture treatment, where acetate was used as an electron donor (*K*_m_ = 35), although at a slightly higher temperature of 37 °C [38], suggesting a reasonably good co-treatment process. Most published studies (Table 2) presented *V*_max_ and *K*_m_ values for either acetate or ethanol oxidation at mesophilic SRB cultures. As such, it is difficult to make a direct performance comparison with the current study.

**Table 2:** Estimated kinetic parameters for COD oxidation by sulfate-reducing enrichment cultures.

Source of inoculum	Temp. (°C)	Carbon source	*K*_m_ (mg/l)	Reference
AMD and MWW mixture	20	AMD and MWW	4.3	[8]
SRB and methanogens	30	Acetate, butyrate	9.5	[39]
Enriched SRB culture	31	Acetic acid	5.9	[15]
Granular sludge	35	Acetate	2.7–3.5	[40]
*Desulforhabdus amnigenus*	37	Acetate	35	[38]
*Desulfobacter postgatei*	30	Lactate	3.8–4.5	[37]
*Desulfobacca acetoxidans*	35	Acetate	35	[38]
Granular sludge (mining)	35	Ethanol	4.3–7.1	[40]
AMD and HWW mixture	34	HWW	7.3	This study

The sulfide inhibition of a culture of enrichment that reduces sulfate was well established in a noncompetitive inhibition model (Fig. 6c). The correlation between sulfide concentration and COD use was not linear, and the use of metals (Fe and Zn) with examined concentrations was not completely inhibited. COD substrate use is correspondingly impaired at concentrations of zinc and iron greater than 8 and 24 mg/l (Fig. 6d). The dissolved sulfide inhibition constants (*K*_i_) were calculated to be 3.6 mg/l for the associated growth, and the related *K*_i_ value was 9 mg/l for H_2_S.

### 3.3 Factors affecting the COD oxidation kinetics

This study realized an optimal pH range of 6.1–8.2 for sulfate-reducing bacteria in AMD/HWW reactor co-treatment. One of the critical factors in microbial reactions is redox potential for controlling the outcome of key chemical elements like sulfur and iron. The findings agreed with Smyntek et al. [41] that the biochemical process in this experiment comprises microbially mediated oxidation with Fe^3+^ and sulfate of the labile fraction of the organic matter. Dissolved oxygen was measured to be < 0.01 mg/l, and the oxidation–reduction potential measured in a range of −65 to −198 mV. Under these conditions, iron occurs as Fe^2+^ and sulfur as S(II), leading to iron sulfide formation.

The precipitation of zinc and phosphates was observed in this study following a similar removal system trend in wastewater treatment [33,42]. Iron concentrations decreased during the initial phase of the experiments after HWW and AMD were mixed in the reactor, confirming Fe precipitation with phosphate or hydroxides, similar to several studies’ observation [3,9]. The iron (III) reducing bacteria contending with SRB may have caused by iron inhibitive effect demonstrated within high Fe concentrations. Deng et al. [8] found a similar inhibitive effect trend of Fe(III) for co-treatment with MWW and AMD. For this current work, in the experiment, the total iron exhibited a significant decrease from 44 to <±2 mg/l between day one and day 30 (One-way ANOVA, *F* = 19.21 *P* < 0.001). The effluent samples from the reactors could not measure copper and aluminum, but manganese and magnesium were measures at < 2 ± 0.7 mg/l. Hence there is no report on these chemical elements and were excluded from the inhibitive model. The AMD/HWW mixing ratio was controlled in the first phase of treatment to prevent SRB's inhibitive consequences.

### 3.4 Toxicity assessment

The toxicity results on both standardized tests with V. *fischeri* show EC_50_ (at 5 min) is higher than 50% of effluent for all samples. This result demonstrates that the assay (at 5 min) are non-toxic. Nevertheless, the results for EC_50_ (at 30 min) were different from EC_50_ (at 5 min), where the results were more significant than 3 TU. The concentrations ranged from 4.4–5.8, which shows that the FBR's effluent toxicity on V. *fischeri* is similar to municipal wastewater toxicity.

**Fig. 7:**
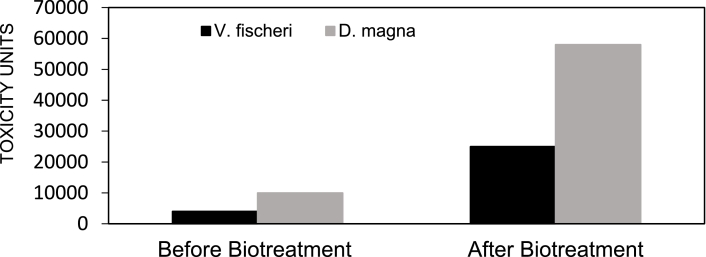
Toxicity of the HWW and AMD sludge before and after the treatment in the FBR.

On the other hand, toxicity EC50 values obtained from D. *magna* biological assays were higher than 2 TU but still categorized as low toxicity acute risk. The concentrations ranged from 15 to 112 TU for EC_50_ on the effluent. The toxicity values before and after biotreatment in the FBR are shown in Fig. 7. The toxicity of effluent from the FBR could hurt the aquatic organisms. These toxicity values do not correlate with the NH_4_^+^ values (34±12 mg/l) measured in the FBR effluent samples; thus, a few areas of concern were identified. Since the HWW is mixed with the AMD (highly acidic), it may result in the development of intermediate degradation agents with higher polarity and toxicity than the original compounds [43]. The

During the ecotoxicity testing process, the effluent sample pH was corrected to ± 7.4 using NaOH, causing some uncertainty to the results. On the contrary, some aqueous-phase degradation studies have observed a similar trend, where the toxicity increased at the end of the treatment for some micropollutants, like carbamazepine and ibuprofen [43]. As such, in this process, ibuprofen was present, with its removal assessed.

### 3.5 The microbial diversity in the fluidized-bed reactor

The bacterial communities were maintained for over 120 days in the FBR, then characterized by culture-independent molecular methods [1] and the development of bacterial culture [22]. A 16S rRNA gene and dsrA gene-based clone library was used to assess the microbial diversity, and results suggested that most functional group diversity in the FBR was sulfate reducers and fermenters. In the fermentative group, sulfate-reducing bacteria species were abundant (>90% related) *Desulfobacter, Desulfococcus, Desulfomona, Desulfomicrobium, Desulfovibrio* [44], and *Syntrophobacter*. Also, a few other bacterial species were present (>94% related) in the domain of *Nitrospira, Clostridia, Crenarchaeota*, and *Thermodesulfobacteria* [45]*.* Church et al. [46] found the involvement of SRB community like *Desulfosporosinus, Bacillus*, and *Clostridium* in sulfate reduction process. Another study by Hiibel et al. [47] compared the microbial diversity in two field-scale pilot SRB bioreactors used to treat acidic water. The results demonstrated a 60% reduction in sulfate from one reactor. Moreover, the study identified *Desulfovibrio* taxa, and the clone library confirmed that the enhanced sulfate reduction was due to the availability of SRB (*Desulfovibrio, Desulfobulbus*, and *Desulfosporosinus*). Elsewhere, different organic reactive mixtures were assessed on AMD remediation with consideration of HRT on performance, organic substrates, and microbial communities [23]. These studies found that organic mixtures achieved > 70% SO_4_^2−^ reduction, and > 99% Fe and Zn removal. The dominance of the microbial community was from the SRB belonging to genera *Desulfobacter, Desulfomona, Desulfobulbus, Desulfomicrobium, Desulfovibrio, Desulfococcus*, and other fermentative groups.

The *Clostridia* can convert trichloroethene to ethane, but also capable of fermenting organics to sugars in anaerobic digesters [48]. *Clostridium* species' presence indicates that the biotreatment system can treat high salinity wastewater [8]. The other strains were affiliated with *Prolixibacter bellariivorans* with more nitrate-reducing bacteria, which can grow at very low temperatures, < 4 °C [49]. These species' existence confirms that the sulfidogenic FBR can treat acidic, sulfate, and nutrient-rich wastewater at mesophilic conditions. The presence of *Leptolinea tardivitalis* further presents a possible co-existence of sulfidogenic bacteria and methanogenic [50], however, the sulfidogenic bacteria remain dominant. The COD/SO_4_^2−^ ratio, pH values, and redox potential conditions, affect the development of diverse community, but the increase of microbial diversity stabilizes the biofilm under fluctuating conditions [47,51]. In this study, the COD/SO_4_^2−^ ratio was controlled at approximately 0.67 which evidently affected the development of the microbial community, when compared the a ratio of 2 that was used by Deng et al. [8].

Fifteen operational taxonomic units were found in the sulfidogenic fluidized-bed reactor library, and distribution of phylogenetic groups is presented in Table 3. The DGGE evaluation was conducted to assess the dynamics in the microbial communities of the FBR. The average anaerobic microbial diversity was observed not to yield the pure strains of the significant genotypes in the DGGE study of the *Magnetobacterium* genera [22]. The reason could be the challenge in achieving pure strains of anaerobes during the FBR enrichment stage. It is advised that culturing and molecular methods are combined to assess more species in the sulfidogenic FBR microbial community [22]. However, the FBR had many sulfate-reducing species of various genera.

**Table 3:** The gene clone's distribution and operational taxonomic units (OTU) in the FBR with denaturing gradient gel electrophoresis (DGGE).

Phylogenetic group	Library		DGGE
	% of clones (*n* = 85)	OTUs	OTUs
*Gamma proteobacteria*	2.1	1	2
*Delta proteobacteria*	52.4	5	4
*Beta proteobacteria*	1.7	1	1
*Chloroflexi*	4.7	1	1
*Clostridium*	3.1	3	1
*Magnetobacterium*	–	–	1
*Nitrospira*	34.6	3	2
*Desulfomicrobium*	1.4	1	1
Undefined	–	–	1
Total	100	15	14

The co-treatment strategy combines the sulfur, carbon, and nitrogen cycles into a single system for wastewater treatment. Sulfidogenes, not like methanogens, may use a wide range of substrates at variety of conditions, like temperature range (10–45 °C). Therefore, reasonable conditions of acidophilic, thermophilic, and neutrophilic SRB can treat wastewaters with pH 4–9 [14,52]. This would encourage the use of anaerobic treatment for a variety of sulfate-rich ground and hospital wastewaters.

### 3.6 Mass balance of COD and sulfur

Estimated sulfate reduction rates based on COD oxidation were higher than the measured rates, which agrees with Sahinkaya et al. [16], that some of the electrons were most likely not utilized for sulfate reduction. During continuous experimental mode, sulfate reduction and COD oxidation decreased dissolved sulfide concentration at HRT less than eight hours. The sulfate, Fe and Zn reduced mass quantities by oxidized COD were calculated 1.2 g SO_4_^2−^/g COD, 6 g Zn/g COD, and 9.5 g Fe/g COD. At an HRT of 12 h, about 540 mg/l/d Fe and 490 mg/l/d Zn concentrations of effluent dissolved Fe and Zn remaining under 0.01 mg/l in the effluent stream. Fermentative reactions influenced some of the electron flow; detected *Clostridium* confirms this as a microbial community member in the FBR. Also, there was less evidence of methanogenesis assays in the microbial community, which confirms no methane production in the FBR. Mass balance evaluation estimated approximately 50±9% of total sulfur, of which 10±3% is dissolved sulfur in the sludge, and approximately 50% of sulfur can be accounted for in metal precipitation.

### 3.7 Pharmaceutical removal

The analytical method was validated by the determination of SPE efficiency, which was higher than 80% for all compounds, and the linearity was calculated to be r^2^ 0.96. The removal efficiencies for all anti-inflammatories group assessed did not demonstrate excellent results at the end of treatment (Table 4). In a sulfidogenic treatment system, it was expected that the microorganisms capable of degrading some organic compounds could do the same for the selected pharmaceuticals. For example, ibuprofen has shown bacteria degradation in some studies [7,53].

**Table 4:** Pharmaceutical's concentration in the influent and effluent samples of the FBR.

Compounds	Influent		Effluent		% Removal
	Concentration (ng g^−1^)	Relative Std. Deviation%	Concentration (ng g^−1^)	Relative Std. Deviation%	
Diclofenac	277	4.12	132	0.91	52.3
Ibuprofen	203	13.3	84	0.68	58.6
Ketoprofen	39.1	5.87	22.7	1.05	41.9
Naproxen	28.1	1.12	15	0.21	46.6

The ibuprofen and diclofenac compounds achieved the highest removal rates in the sulfidogenic FBR of 58.6 and 52.3%. An anaerobic analysis on a laboratory scale showed 30–60% ibuprofen elimination under anoxic conditions and greater than 75% diclofenac degradation [54]. As such, the results of this study are not far off from what other studies have achieved. Moreover, diclofenac is one of the most used anti-inflammatories and has proved to be less removed in traditional wastewater treatment plant (WWTP) [55]. In previous studies, WWTP achieved a 50–65% removal of ketoprofen and naproxen [56]. Unfortunately, this study did not achieve comparable removal results demonstrated by these studies. Table 4 shows the removal rate for ketoprofen and naproxen of 41.9% and 46.6%, respectively.

These results should be viewed with the understanding of the allowable pharmaceutical discharge limits as pollutants to the environment. Without a doubt, ineffective removal efficiency of pharmaceuticals in WWTP leads to the degradation of surface water, ground water and drinking water quality [57]. In well-developed economies like the United State of America, Canada and other European countries, there are strict regulations that control the disposal of pharmaceuticals into the sewage [58]. However, in the context of South Africa, there are still no legislative measures in place regulating the discharge of pharmaceutical residues in water bodies. It is anticipated that more stringent measures will be in place soon, as such monitoring of these environmental stressors is important in the field.

## 4 Conclusions

The study provides important information regarding the performance of sulfidogenic bioreactors treating acidic metal-containing water and hospital wastewater. This work demonstrates the possibility of an SBR reactor for synchronized decreasing concentrations of COD, metals, sulfate, and selected anti-inflammatory pharmaceutical compounds in wastewater. At an HRT of 12 h, about 540 mg/l/d Fe and 490 mg/l/d Zn were precipitated with effluent soluble Fe and Zn concentrations remaining below 0.01 mg/l in effluent stream. The wastewater pH was increased from 2.5 to 8.2 during the co-treatment. Michaelis Menten constants (*K*_m_) for COD oxidation found in the batch FBR were 7.3 mg/l. The maximum oxidation velocity (*V*_max_) was found to be 0.12 mg/l min. The dissolved sulfide inhibition constants (*K*_i_) were 3.6 mg/l for the associated growth, and the related *K*_i_ value was 9 mg/l for H_2_S. The microbial community provided insights into the main microbes in biological treatment. The dominant species in the treatment process belong to the *Proteobacteria* group (especially *Deltaproteobacteria*). The results from this study seem to provide baseline for further research to develop water treatment technologies further to inform municipal regulations and legislations.

## Declaration of Competing Interest

The author(s) declare that there is no conflict of interest
